# The Double-Edged Sword Effect of Relational Crafting on Job Well-Being

**DOI:** 10.3389/fpsyg.2022.713737

**Published:** 2022-02-16

**Authors:** Shanshan Li, Bin Meng, Qingjin Wang

**Affiliations:** ^1^Department of Industrial Economics, University of Chinese Academy of Social Sciences, Beijing, China; ^2^Department of Economics and Management, Hengshui University, Hengshui, China; ^3^Business School, Qingdao University, Qingdao, China

**Keywords:** relational crafting, job well-being, work load, supervisor-subordinate *guanxi*, double-edged sword effect, job demands-resources model, conservation of resources theory

## Abstract

Is relational crafting always beneficial? Despite the increasing research on the positive outcomes of relational crafting, some evidence still indicates its dysfunctional consequences. The current study proposed a double-edged sword effect of relational crafting on job well-being, including work dynamics and emotional exhaustion, with an integrative perspective from the resource loss and resource acquisition perspectives based on the job demands-resources model and the conservation of resources theory. By conducting a two-stage questionnaire survey on 323 employees, the results demonstrate that: (1) On the one hand, relational crafting induces emotional exhaustion through increased work load; (2) On the other hand, relational crafting also displays positive effect on increasing work dynamics and decreasing emotional exhaustion by fostering supervisor-subordinate *guanxi*. By analyzing the double-edged sword effect of subordinates’ relational crafting on job well-being from the two processes of resource loss and resource acquisition effects, a more complete influencing mechanism between relational crafting and job well-being is constructed, which improves the understanding of relational crafting, enriches the literature on proactive behavior and provides a more integrated theoretical basis for researchers and managers.

## Introduction

With the development of society, economy, science, and technology, the nature of work has become vaguer and more complex (Womack, 2018), requiring employees to adapt to dynamic jobs effectively (Slemp and Vella-Brodrick, 2013). Accordingly, the normative job description no longer applies to current workplace shifts, and a new form of flexible job redesign, referred to as job crafting, has been suggested (Wrzesniewski and Dutton, 2001). As a proactive employee behavior, job crafting generally encompasses idiosyncratic changes to their tasks (task crafting), relationships (relational crafting), and cognition (cognitive crafting) (Wrzesniewski and Dutton, 2001). With the progress of studies, job crafting scholars have indicated different crafting orientations, such as approach avoidance or promotion prevention (e.g., Bruning and Campion, 2018; Zhang and Parker, 2019). Compared with promotion prevention, we agree with the view of Zhang and Parker (2019) that the approach-avoidance perspective is more direct. Evidence from research suggests that “job crafting is characterized more by effortful and directed actions to seek positive aspects of work rather than by withdraw-oriented behaviors concerning the negative ones” (Costantini et al., 2021, p. 336). Thus, we distinguish approach crafting from avoidance crafting and focus on the former, which is intended to serve employees by creating positive psychological state and favorable job characteristics for them, which distinguishes the process from other forms of job crafting (Wrzesniewski and Dutton, 2001; Tims et al., 2012; Bruning and Campion, 2018). Furthermore, relational crafting has higher practical significance for employees in Chinese organizations, as China attaches great importance to workplace *guanxi* under the influence of the Confucian culture (Chen et al., 2009; Tang et al., 2020). *Guanxi* is “an informal, particularistic personal connection between two individuals” (Chen and Chen, 2004, p. 306). Workplace *guanxi* is necessary and is associated with positive outcomes, such as preferential decisions (Wei et al., 2010) and higher job satisfaction (Cheung et al., 2009). Meanwhile, positive workplace *guanxi* can be developed and established by affection and reciprocal exchange (Zhai et al., 2013). Relational crafting depicts employees’ behaviors to change relational boundaries, involving activities of seeking, building, and/or maintaining better relationships with preferred individuals in the workplace (Bruning and Campion, 2018). This behavior has been found to help employees have more supportive and rewarding interactions, resulting in various positive outcomes (Jutengren et al., 2020). Relational crafting can be categorized as approach oriented and avoidance oriented. In our study, we focus on approach-oriented relational crafting. Therefore, when discussing relational crafting in the paper, it relates only to approach-oriented relational crafting. We also believe that further studies on relational crafting will be a meaningful and important topic in the contemporary Chinese context.

Although the concept of relational crafting and related research has received wide attention in the past 20 years, most studies examine relational crafting as an element of job crafting, analyzing its outcomes (Lee and Lee, 2018). Recently, various researchers have increasingly turned their attention to the effect of specific crafting forms, such as task crafting and relational crafting, based on the given situation instead of focusing solely on job crafting, suggesting that the results may be more nuanced and accurate if a form of crafting is investigated separately (Dierdorff and Jensen, 2017; Teng et al., 2020; Geldenhuys et al., 2021). Existing literature has predominantly centered on the positive effects of relational crafting, for which several main reasons have been proposed. For example, relational crafting is thought to help employees cultivate job meaningfulness (Michaelson et al., 2014), enhance demand-supply fit (Lu et al., 2014), and improve work adaptability (Rofcanin et al., 2019) while facilitating job performance (Geldenhuys et al., 2021). However, to date, direct examinations of the possible negative effects of relational crafting, such as increased work load, have remained absent. Nevertheless, some scholars have begun to indicate that pro-self-focused proactive behaviors are also associated with increased levels of fatigue and reduced job well-being (Berg et al., 2010; Weseler and Niessen, 2016). For example, Strauss et al. (2017) argued that personal initiative is associated with job strain due to resource depletion. Zacher et al. (2019) further examined the negative impact of personal initiative on occupational well-being (emotional engagement and emotional exhaustion), suggesting that it can cause a negative shift in employee’s mood. Moreover, some studies have also observed that crafting can cause a negative effect due to the intermittent feelings of regret and increased stress and conflict (Wrzesniewski and Dutton, 2001; Berg et al., 2010; Dierdorff and Jensen, 2017). Therefore, as a specific proactive behavior and a specific form of job crafting, how are these conflicting viewpoints and empirical evidence reflected in subordinates’ relational crafting and how does relational crafting affect job well-being?

Drawing on the job demands-resources model (JD-R model) and the conservation of resources theory (COR theory), the present study aims to offer a more comprehensive understanding of relational crafting. In particular, we postulate that relational crafting has a double-edged sword effect on job well-being based on the supervisor-subordinate context. Using this argument, we further explain the underlying mechanisms of the effects of relational crafting on job well-being from two perspectives of resource loss and resource acquisition, in accordance with the JD-R model and the COR theory. The resource loss path indicates that relational crafting is negatively related to job well-being by increasing work load. As an extra-role behavior, relational crafting consumes massive psychological and cognitive resources from crafters (i.e., subordinates), thereby creating excessive stress and lowering job well-being. When subordinates craft their jobs by extending social relationships at work, they might be ostracized by others because of the substantial change they cause to the existing *guanxi* circle, which in turn might bring pressure and negatively impact job well-being. Therefore, work load can lead to a health impairment process and, in turn, negatively affect subordinates’ job well-being. The resource acquisition path suggests that relational crafting is positively related to job well-being by improving supervisor-subordinate *guanxi* (SSG). Relational crafting is a process in which individuals adjust their social relationships to enhance their social bonds (Wrzesniewski and Dutton, 2001; Nielsen and Abildgaard, 2012). By seeking support, feedback, and guidance from supervisors, and by actively concerning, caring for, and assisting supervisors, subordinates can increase work efficiency, improve communication quality, boost relatedness with supervisors (Rudolph et al., 2017), and obtain positive responses from them, thereby establishing a better SSG. A higher quality of SSG will inevitably enhance job well-being. Therefore, SSG can lead to a motivational process and, in turn, positively affect subordinates’ job well-being.

To advance the relational crafting research, we investigated both its negative and positive indirect effects on job well-being, including work dynamics and emotional exhaustion. This study makes four main contributions to the literature: First, prior studies on the impact of relational crafting always consider it a part of job crafting (Slemp and Vella-Brodrick, 2014; Slemp et al., 2015; Lee and Lee, 2018). We aim to make a more nuanced examination of relational crafting consequences. Therefore, we focus on the relationship between relational crafting and job well-being to extend the related research based on the Chinese context, where *guanxi* and friendship are highly emphasized. Thus, our study improves the understanding of relational crafting and enriches the literature on job crafting. Second, although studies on relational crafting’s impact on attitude-related outcomes have attracted much attention (Michaelson et al., 2014; Jutengren et al., 2020), few scholars have analyzed its positive effect from the context of the SSG perspective. We investigated whether subordinates’ relational crafting could influence their job well-being by affecting SSG. In this way, our study extends over two domains, job crafting literature and social network literature, contributing to job well-being; namely, work dynamics and emotional exhaustion. Third, although past research provides burgeoning evidence of the positive consequences of relational crafting, we suggest that its outcomes may be more varied (Wrzesniewski and Dutton, 2001; Weseler and Niessen, 2016). We argue that relational crafting is not only associated with attitude-related positive outcomes but also has the potential to cause high work load harmful to subordinates’ job well-being. Hence, we propose that relational crafting can be a “double-edged sword” and may lead to lower job well-being, which supplements and expands the existent research on proactive behavior. Finally, previous studies on relational crafting are mainly based on the self-determination theory and the JD-R model from a single perspective. This study integrates the JD-R model and the COR theory from the resource loss and the resource acquisition processes to fill this gap, providing a new theoretical explanation for the connection between proactive behavior and job well-being.

## Theory and Hypotheses

### Theoretical Foundations of Relational Crafting and Job Well-Being

Relational crafting is a type of job crafting. Wrzesniewski and Dutton (2001) proposed a widely applied definition of job crafting by regarding it as an employee behavior that actively changes role boundaries. On this basis, they also classified three types of job crafting: task crafting, relational crafting, and cognitive crafting. However, in recent years, with increasing attention on the other effects of job crafting (apart from the positive ones), more comprehensive classifications have been suggested. For example, Weseler and Niessen (2016) divided job crafting into five dimensions, combining the crafting direction (expansion reduction) with crafting content (task relationship) while retaining cognitive crafting. Bruning and Campion (2018) built role-resource approach-avoidance taxonomy to divide job crafting into four dimensions: approach role/resource crafting and avoidance role/resource crafting. Lichtenthaler and Fischbach (2019) categorized it into promotion-focused and prevention-focused job crafting based on the regulatory focus theory. Zhang and Parker (2019) defined job crafting as a hierarchical structure after affirming the importance of approach-avoidance motivation. They viewed crafting direction (approach avoidance) as the first level, crafting content (behavior cognition) as the second level, and crafting goals (resources demands) as the third level, thereby forming an integrated crafting model with eight dimensions. In light of previous related research outcomes and Zhang and Parker’s (2019) view, we believe that the approach-avoidance perspective is more appropriate. Approach crafting refers to positive, goal-oriented, and problem-oriented crafting behaviors, while avoidance crafting involves behaviors with negative and evasive aspects (Costantini et al., 2021). Accordingly, the key implication arising from the achievements above is that relational crafting also has approach-avoidance orientations with the characteristics corresponding to approach-avoidance job crafting. In the current study, as aforementioned, we focus predominantly on approach-oriented relational crafting.

The concept of job well-being was proposed to describe the specific expression of well-being in work, that is, the perceived well-being of employees in their workplace. It can be used to understand employees’ cognitive evaluations and affective experiences (Diener, 2000; Li et al., 2021). In terms of cognitive evaluation, job well-being is reflected in the evaluation of overall job satisfaction. For example, Ryff and Keyes (1995) pointed out that the nature of job well-being includes employees’ evaluations of job autonomy, environmental mastery, and personal growth. Regarding affective experience, job well-being is perceived as the balance between positive and negative effects. Compared with the cognitive dimension, the affective dimension not only has a more significant positive effect on subordinates’ work behaviors and performance but also reflects better their psychological and emotional changes in the process of relational crafting. Staw et al. (1994) argued that employees with stronger positive affect are more likely to receive positive comments from their supervisor. Arnold et al. (2007) also found that affective well-being can explain job performance variation after the influence of fixed control variables of job satisfaction and organizational commitment. In addition, Chinese subordinates’ relational crafting may require them to invest more physical and psychological resources to deal with the vastly rich and complex *guanxi*. Receiving support from supervisors can act as an energy supplement for subordinates. Therefore, we emphasize the affective experience of job well-being. As Bradburn (1969) proposed, “the positive affect and negative affect are the two independent dimensions of job well-being, and when the frequency of positive affect is higher than that of negative affect, employees will exhibit job well-being.” Diener (2000) stated that “job well-being is a kind of affective experience in which positive affect (e.g., happiness, joyousness, enthusiasm, etc.) surpasses negative affect (e.g., shame, anxiety, depression, etc.) and occupies the dominant position.” We, therefore, pay attention to the positive and negative aspects of job well-being. Based on the studies of Cropanzano and Wright (2001) and Du et al. (2014), we consider work dynamics and emotional exhaustion as the embodiment of the positive affect, while the negative affect measures job well-being from the opposite direction. Work dynamics include employees’ spiritual attitudes with vitality and vigor. Ryan and Deci (2001) believed that employees feeling true well-being are full of vitality and dynamics, making work dynamics a key indicator of job well-being. Emotional exhaustion is the core feature of burnout, as it describes a feeling and state of emotional draining caused by individuals’ personal resources being nearly drained by work stressors (Maslach and Jackson, 1981). Warr (1987) proposed that emotional exhaustion is also a dimension of job well-being.

### Theoretical Background

Relational crafting has opposite directional influences on job well-being, and the JD-R model and the COR theory provide a theoretically integrated framework, detailing when relational crafting damages or benefits crafters’ well-being. The JD-R model, proposed by Demerouti et al. (2001), categorizes every occupation’s characteristic into general categories: job demands and job resources. Job demands refer to the “negative factors” that require an individual’s sustained physical and/or psychological effort or skills in the job, which are more associated with physiological and/or psychological costs, including high work pressure, interpersonal conflict, job insecurity, and an unfavorable physical environment (Demerouti and Bakker, 2011). Job resources refer to the “positive factors” with motivational potential at the organizational, interpersonal, and task level to help employees achieve work goal, reduce job demands and the associated physiological and psychological costs, while stimulating personal growth and development, including job security, leader support, and job autonomy (Demerouti et al., 2001; Demerouti and Bakker, 2011; Bakker and Sanz-Vergel, 2013). The COR theory, proposed by Hobfoll (1989), indicates that employees with abundant resources have more opportunities to obtain additional resources and gain benefits from them, whereas those who lack vital resources are more likely to experience subsequent losses and perceive threats (e.g., stress) (Hobfoll, 1989). According to the COR theory, one of the basic needs of human beings is to acquire and accumulate resources to conserve other important resources that are crucial for obtaining higher-level goals or an ideal future state (Hobfoll, 2011; Hobfoll et al., 2018). Meanwhile, the COR theory teaches us that when individuals’ access to essential resources is threatened, when they lose vital resources, and when fewer resources offset resources loss, they may experience stress. Resource loss is more striking than resource gain as it constitutes a significant risk to subsistence and impacts people more swiftly (Ojo et al., 2021). Consequently, the core hypotheses of “dual paths” are developed based on the JD-R model and the COR theory, indicating that two different underlying processes play a role in relational crafting. The first is the effect of the resource loss process, suggesting that demanding jobs or ones with chronic or high demands and low personal and job resources may lead to the depletion of energy (i.e., emotional exhaustion) and health problems. The second is the effect of the resource acquisition process, implying that personal and job resources may play an intrinsic motivational role and lead to high job engagement and job well-being (Deng et al., 2021). Therefore, there are both resource loss and resource acquisition effects in relational crafting. The loss effect path depicts negative outcomes, such as health problems and emotional exhaustion, whereas the acquisition effect path describes positive outcomes, such as high job engagement and positive affect.

Based on the JD-R model and the COR theory, on the one hand, we consider work load and elaborate its mediating role in the resource loss path. The process of subordinates’ relational crafting consumes substantial time and energy, which undoubtedly increases work load. Furthermore, subordinates may struggle to deal with the increasingly complex and varied *guanxi*, which will aggravate work load, cause emotional exhaustion and job burnout, and hamper job well-being. On the other hand, we consider SSG and elaborate its mediating role in the resource acquisition path. *Guanxi* is not only an indigenous Chinese construct but also plays an important role in Chinese culture (Miao et al., 2020). SSG, a special type of *guanxi* in Chinese organizations, covers both work-related exchanges and informal and non-work-related interactions between supervisors and their subordinates (Zhang et al., 2014; Miao et al., 2020). When subordinates craft their jobs by deepening relationships (e.g., building new *guanxi*, reconstructing existing *guanxi*, and adapting to *guanxi* with co-workers or supervisors) at work, they build a friendlier *guanxi* with their co-workers and supervisors due to positive self-presentation (Daniels et al., 2014). In particular, due to the bureaucratic consciousness in the Chinese workplace and the fact that supervisors have considerable latitude to make decisions without sticking to formal rules (Miao et al., 2020), subordinates prefer to improve *guanxi* with their supervisor. Thus, in this study, we focus on enhancing the quality of SSG through relational crafting. The high quality of SSG enables subordinates to obtain more personal and job resources, such as emotional support, trust, information, and empowerment from their supervisor (Cheung et al., 2009), thereby increasing work dynamics and promoting job well-being. In sum, we aim to examine whether subordinates’ relational crafting can affect job well-being through work load and SSG.

### Resource Loss Path: The Mediating Role of Work Load

Work load is one of the characteristics/sources of job stress, which can be regarded as employees’ or subordinates’ subjective judgment of workload (Spector and Jex, 1998; Price, 2001). As previously reported, work load is mainly reflected by long work time, fast work pace, and a large number of assigned tasks (Spector and Jex, 1998). Relational crafting consumes resources and causes work load increase. According to the COR theory, we believe that work load is a typical representative of the resource loss path, and subordinates’ relational crafting will increase their work load as they have to divert some of the time, energy, and resources, which they normally spend on their own job to create an expanded and deepened social and work *guanxi*, thereby negatively influencing their work pace since personal resources are finite and “travel in packs, or caravans.” (Hobfoll, 2011; Hobfoll et al., 2018). Meanwhile, the consumption of relationships and tasks on subordinates’ personal and job resources may cause a lack of sufficient cognitive resources to enact other formally prescribed behaviors and to fulfill others’ role expectations if fewer resources offset these resources loss (Hobfoll et al., 2018), resulting in increased stress and work load. In addition, when subordinates craft relationships, they may be involved in a series of complex intellectual activities that can take up more time, energy, cognitive ability, and other resources because of the complicated *guanxi* in the Chinese workplace (Luo et al., 2016). As a result, subordinates feel overwhelmed. Taken collectively, subordinates’ relational crafting can inevitably and significantly cause work load increase if the resource loss cannot be replaced in time.

Furthermore, work load is an important source of job demands for subordinates, impacting their psychology (Ilies et al., 2010). Building on the JD-R model, job demands are the physical, psychological, and social requirements of the individuals, which require effort and cost. When job demands are consistently high and no job resources are available to compensate, the individuals’ energy will be constantly depleted, which may lead to energy exhaustion (Demerouti et al., 2001). The COR theory also states that when individuals perceive constant resource consumption and face the threat of resource loss and the failure to obtain the corresponding return on resource investment, they tend to become more sensitive to resource reduction, resulting in undesirable results, including reduced job well-being (Huang et al., 2019). As previously proposed, the resource occupation of subordinates’ relational crafting will inevitably and significantly reduce their resources to enact other formally prescribed behaviors as resources are finite, and thus increase their subjective job stress, work pace, and perceived assignments, thereby enhancing work load and reducing well-being. Prior research also indicated that excessive work load not only had a negative effect on job status and job satisfaction (Kunte et al., 2017; Mittal and Bhakar, 2018; Hwang et al., 2020) but was also positively related to job burnout and emotional exhaustion (Weigl et al., 2016; Buruck et al., 2020). Therefore, we hypothesize the following:

Hypothesis 1a: Relational crafting is negatively related to work dynamics by increasing work load.
Hypothesis 1b: Relational crafting is positively related to emotional exhaustion by increasing work load.

### Resource Acquisition Path: The Mediating Role of Supervisor-Subordinate *Guanxi*

Supervisor-subordinate *guanxi*, a specific and essential type of *guanxi* in the Chinese workplace, is defined as “the relationship between a subordinate and his or her immediate supervisor,” creating a sense of “social connections” based on mutual interest and benefit (Wong et al., 2003, p. 484). SSG researchers view subordinates as a vital part in determining the quality of SSG (Miao et al., 2020). Prior literature has shown that supervisors in Chinese organizations always offer different bonuses and opportunities to their subordinates based on *guanxi* (Chen et al., 2009), which motivates subordinates to invest in their *guanxi* with supervisors. This implies that subordinates who undertake relational crafting in Chinese organizations will attach greater importance to improve the *guanxi* with their supervisor instead of this with co-workers (Yin et al., 2017), which enables them to understand better their supervisor’s demands and preferences and gain the supervisor’s trust, in a way becoming a member of the supervisor’s “in-group” (Lam et al., 2015). Moreover, subordinates who craft their interpersonal relationships at work by choosing to spending more time with valued, liked, and preferred individuals can establish a *guanxi* circle in line with their preference, thereby enhancing job engagement (Yin et al., 2017). Increased job engagement demonstrates the positive image to their supervisor. This relational crafting represents a proactive impression management tactic that can change supervisors’ cognition and make a good impression on them that would obtain positive responses (Fuller et al., 2012). Meanwhile, relational crafting can be perceived by supervisors as a pro-organizational and initiative behavior that contributes to an organization, and the subordinates will, in turn, become closer than others to a member of the “in-group.” The identity of an “in-group” member means that subordinates’ relational crafting not only establishes a strong working relationship with their supervisor but also allows to keep in touch with the supervisor, resulting in improved private relationship, since an “in-group” member would be more valued and favored by his or her supervisor (Xu et al., 2019). For example, the supervisor may give the subordinates (i.e., crafters) more authorization and a high-performance rating formally, and may also put more effort in the informal relationship, including visiting each other or eating dinner together after work. Altogether, relational crafting has a positive spillover effect for the *guanxi* between subordinates and their supervisor both in and outside the workplace (Chen et al., 2011). High quality of working and private relationships is an important foundation for high quality of SSG as it is not limited to the scope of work (Chen et al., 2009; Tang et al., 2020). Furthermore, as Yin et al. (2017) argued, the relational resources accumulated through subordinates’ relational crafting not only create opportunities for task crafting but also change their supervisor’s attitude toward such behaviors, which, in turn, enables them to assist supervisors in jobs by extending task boundaries and taking on additional tasks on the basis of easing supervisors’ worries about their extending task crafting (e.g., perceived threat). In other words, by taking on extra-role responsibilities based on relational crafting, subordinates’ loyalty and affinity will be highly valued, which is the key to establishing and maintaining high quality of SSG (Tang et al., 2020). According to the COR theory, subordinates’ relational crafting is also a behavior to acquire and accumulate resources, which increases their core *guanxi* resources in the Chinese workplace. Hence, we posit that relational crafting improves SSG.

Due to the differential pattern in Chinese organizations, supervisors always give different opportunities and resources to their subordinates according to the quality and relatedness of SSG. High quality of SSG is not only an important job resource (Bakker and Demerouti, 2007) but also constitutes a social resource that can spill over into the workplace and improve subordinates’ additional job resources, such as opportunities, support, autonomy, and development (Guan and Frenkel, 2019). Based on the JD-R model and the COR theory, we believe that SSG can improve subordinates’ resource accumulation, enabling them to engage further in a job and accrue more job resources that contribute to higher work dynamics and lower emotional exhaustion (Hobfoll, 1989; Demerouti et al., 2001). Tan et al. (2020) suggested that when employees had a high level of organizational support, their sense of responsibility to an organization increased, creating higher work commitment and work dynamics. Huang et al. (2010) found that SSG was considered a valuable resource that could help overcome emotional exhaustion at work. Furthermore, several scholars have also explained and examined the relationship between SSG and subordinates’ affective outcomes. For example, Zhai et al. (2013) argued that SSG was positively related to job satisfaction. Li et al. (2018) also highlighted the importance of SSG and stated that SSG cultivated subordinates’ happiness through increased resources and personal power. In sum, a key implication arising from the high quality of SSG is that when subordinates have that with their supervisor, they are more likely to sustain positive work mood because of the additional emotional support, resulting in increased work dynamics and reduced emotional exhaustion. Therefore, we hypothesize the following:

Hypothesis 2a: Relational crafting is positively related to work dynamics by increasing SSG.
Hypothesis 2b: Relational crafting is negatively related to emotional exhaustion by increasing SSG.

**FIGURE 1:**
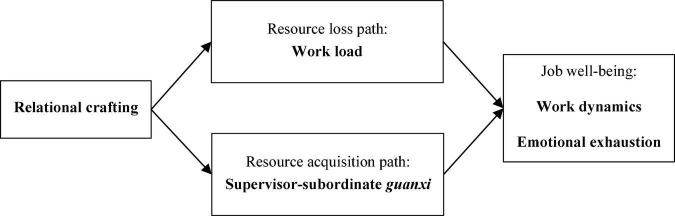
Theoretical model.

Figure 1 shows our theoretical model.

## Materials and Methods

### Sample and Procedure

We aim to explore how relational crafting affects job well-being. Therefore, the sample companies must have a certain degree of openness and flexibility to lay a contextual foundation for crafting activities. A total of 500 employees from high-tech enterprises and hospitals in the eastern coastal areas of China were surveyed with the help of relatives and friends. All the participants were full-time employees and were employed in different occupations, including human resource specialists, nurses, doctors, accountants, and construction managers. Before data collection, several researchers had met with some participants to describe to all the participants the aims, procedures, and relational crafting’s connotation to ensure that everyone could clearly understand all items.

At Time 1 (T1), we sent 500 employees the link to the questionnaire website and asked them to report relational crafting and demographics (gender, age, education, and working years), receiving the completed surveys from 452 participants (response rate: 90.40%). At Time 2 (T2) (about 3 months later), SSG, work load, job well-being, and demographics (gender, age, education, and working years) were measured by sending WeChat messages and e-mails with the questionnaire website link to the respondents at T1. A total of 375 employees returned the T2 survey (response rate: 82.96%). The two-wave data were matched by participants’ WeChat and e-mail. All ratings were anonymous as we did not collect clear names. After deleting the invalid forms, a total of 323 matched surveys were retained (overall response rate: 64.60%). The results of *t*-tests showed that there were no significant differences on demographics or T1 variables (i.e., relational crafting) existed between the T2 responders and non-responders (Dooley and Lindner, 2003). About 55.11% of the participants were male; 25.70% were under the age of 25, 25.38% were aged between 25 and 35, 34.06% were aged between 35 and 45, 14.86% were over 45 years old; 66.87% of the participants had a bachelor’s degree or above; 19.81% had work for a year or less, 39.63% had worked for 2–5 years, 24.15% had worked for 6–9 years. About 42.11% of the participants were human resource specialists, 30.96% worked in a medical position, 17.03% were accountants, 9.91% were construction managers.

### Measures

Unless otherwise noted, responses to all items were measured on seven-point Likert-type scales, ranging from strongly disagree (1) to strongly agree (7).

#### Relational Crafting

Relational crafting was assessed using a five-item scale developed by Slemp and Vella-Brodrick (2013). In their study, the main items of relational crafting scale were related to build harmonious interpersonal relationships and did not include reducing or avoiding the interaction with others, which was in line with the current study. A sample item is “Make an effort to get to know people well at work.” The Cronbach’s α score for the scale was 0.861.

#### Work Load

A five-item scale developed by Spector and Jex (1998) was used to assess work load. A sample item is “My job requires me to finish the task quickly.” The Cronbach’s α score for the scale was 0.841.

#### Supervisor-Subordinate *Guanxi*

Supervisor-subordinate *guanxi* (SSG) was assessed using Wong et al.’s (2003) seven-item scale. Their measure reflected the *guanxi* quality between supervisors and their subordinates, which was suitable for our research. Given the characteristics of the scale and the purpose of reducing respondents’ burden to complete the questionnaire, we combined items 4 and 5 into one item, namely, “My immediate supervisor and I are quite willing to help each other (e.g., finding, moving, or decorating a house).” The six items exhibited high internal consistency. The Cronbach’s α score for the scale was 0.910. A sample item is “I have frequent interactions with my immediate supervisor.”

#### Work Dynamics

This variable was measured using a six-item scale from Schaufeli et al. (2002). A sample item is “When I get up in the morning, I feel like going to work.” The Cronbach’s α score for the scale was 0.844.

#### Emotional Exhaustion

Emotional exhaustion was measured using a Chinese version (Li and Shi, 2003) of Maslach Burnout Inventory (MBI) that assessed emotional exhaustion with five items. It had been proved to have high reliability and validity. A sample item is “Job makes me tired.” The Cronbach’s α score for the scale was 0.878.

#### Control Variables

We collected several demographic variables, including gender, age, education, and working years, as prior literature suggested that compared with men, women might implement relational crafting differently in many aspects, and employees with more experience and a higher level of education tended to engage in fewer crafting behaviors (Tims et al., 2013; Dierdorff and Jensen, 2017). Hence, we controlled them to rule out alternative explanations and to carry out a more reliable test. All the controlled variables were dummy coded. Gender was coded as 1 for the participants who were male and 2 for participants who were female. Age was coded as 1 for the participants who were aged under 25 years old, 2 for participants who were aged between 25 and 35, 3 for the participants who were aged between 35 and 45, and 4 for the participants who were aged over 45 years old. Education was coded as 1 for the participants who had finished a high school education or below, 2 for the participants who had an associate’s degree, 3 for the participants who had a bachelor’s degree, and 4 for the participants who had a postgraduate’s degree. Working years was coded as 1 for the participants who had worked for a year or less, 2 for the participants who had worked for 2–5 years, 3 for the participants who had worked for 6–9 years, and 4 for the participants who had worked for 10 years or more.

### Data Analysis

We used SPSS 22.0 and Amos 23.0 for data analysis. First, Cronbach’s α, composite reliability, and confirmatory factor analyses (CFAs) were conducted to assess the reliability and validity of the key variables. Common method variance (CMV) was also assessed. Second, we used path analysis to evaluate the theoretical model (see Figure 1) and the alternative model (adding the direct path from relational crafting to job well-being based on the theoretical model); thus, we chose the optimal model to examine the hypothesized relationships (Anderson and Gerbing, 1988). Finally, we used the bootstrapping method to test mediation because of its high power (Preacher and Hayes, 2004, 2008).

## Results

### Reliability and Validity

First, before conducting reliability and validity test, we had checked CMV because it is a potential issue in the self-reporting approach research. We used Harmon’ one-factor test by including all of the items of the five variables (i.e., relational crafting, work load, SSG, work dynamics, and emotional exhaustion) to examine CMV in SPSS 22.0. When the first emerging factor accounted for over 50% of the extracted variables’ variance, common method bias was suggested and CMV would be a problem. The results demonstrated that the first emerging factor accounted for 14.83% of the explained variance, indicating that CMV was not a significant problem in the present study.

Second, we calculated Cronbach’s α and composite reliability of relational crafting, work load, SSG, work dynamics, and emotional exhaustion to examine the reliability. As mentioned above and displayed in Table 2, the values of Cronbach’s α and composite reliability were greater than the threshold value of 0.80, demonstrating acceptable reliability.

**TABLE 1:** Results of confirmatory factor analyses.

Models	Variables	*c* ^2^	*df*	*c^2^/df*	RMSEA	CFI	TLI	SRMR
Five-factor model	RC, WL, SSG, WD, EE	846.672	314	2.696	0.061	0.928	0.918	0.059
Four-factor model^1^	RC,WL+SSG,WD, EE	1,135.347	318	3.570	0.097	0.833	0.805	0.095
Four-factor model^2^	RC, WL, SSG, WD+EE	1,008.451	318	3.171	0.104	0.823	0.794	0.088
Three-factor model	RC, WL+SSG, WD+EE	1,229.533	321	3.830	0.105	0.736	0.702	0.100
Two-factor model	RC+WL+SSG, WD+EE	1,844.033	323	5.709	0.164	0.635	0.604	0.159
One-factor model	RC+WL+SSG+WD+EE	2,451.275	324	7.566	0.173	0.548	0.510	0.160

*1 & 2 represents different models of Four-factor model.*

**TABLE 2:** Means, standard deviations, and correlations.

Variables	1	2	3	4	5	6	7	8	9
1. Gender									
2. Age	0.093								
3. Education	–0.025	0.144**							
4. Working years	0.044	0.068	0.122*						
5. RC T1	0.001	–0.005	–0.016	–0.044	**0.807**				
6. WL T2	–0.003	0.033	–0.034	–0.027	0.143**	**0.786**			
7. SSG T2	0.007	0.042	0.017	0.001	0.184**	–0.104	**0.833**		
8. WD T2	–0.005	–0.020	0.118*	0.023	0.288**	–0.109	0.468**	**0.758**	
9. EE T2	0.113*	0.109	–0.028	–0.001	−0.106*	0.564**	−0.201**	−0.140**	**0.820**
Composite reliability					0.903	0.890	0.931	0.888	0.911
*Mean*	1.449	2.381	2.712	2.372	4.959	4.914	5.182	4.596	4.696
*S.D.*	0.498	1.017	0.853	0.984	0.587	1.039	0.768	0.540	0.925

**p < 0.01, *p < 0.05. The bold values are the square roots of AVE.

Finally, we conducted a series of CFAs using Amos 23.0 on the scales, including relational crafting, work load, SSG, work dynamics, and emotional exhaustion, to examine discriminate validity (see Table 1). Results showed that the fit of the five-factor model in which items were loaded on their respective measures was better than any other model (χ^2^/df = 2.696, RMSEA = 0.061, CFI = 0.928, TLI = 0.918, SRMR = 0.059). These results of CFA provided full support for the discriminate validity of our study instruments. We also used the square roots of the average variance extracted (AVE) to further examine the discriminant validity. As shown in Table 2, the square roots of AVE were larger than each construct’s correlation coefficients, ensuring satisfactory discriminant validity.

### Descriptive Statistics and Correlations

Table 2 provides means, standard deviations (S.D.), and correlations among study variables. As anticipated, relational crafting (T1) was positively related to work load (T2) (*r* = 0.143, *p* < 0.01) and positively related to SSG (T2) (*r* = 0.184, *p* < 0.01). Work load (T2) positively related to emotional exhaustion (T2) (*r* = 0.564, *p* < 0.01). SSG (T2) was positively associated with work dynamics (T2) (*r* = 0.468, *p* < 0.01), negatively associated with emotional exhaustion (T2) (*r* = −0.201, *p* < 0.01). These results provide preliminary support for the hypotheses proposed above. We further used path analysis to test the entire model and the hypotheses.

### Hypotheses Testing

**FIGURE 2:**
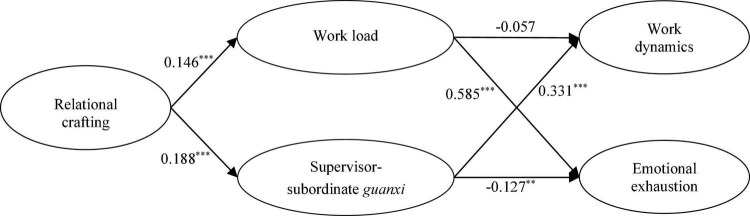
Path analysis results. ***p < 0.001, **p < 0.01, and * *p* < 0.05.

Study hypotheses were tested using path analysis. We added both direct paths from relational crafting to job well-being based on the theoretical model to get the optimal model. Results demonstrated that both the theoretical model (χ^2^/df = 3.146, RMSEA = 0.063, CFI = 0.918, TLI = 0.889, SRMR = 0.066) and the alternative model (χ^2^/df = 3.134, RMSEA = 0.061, CFI = 0.918, TLI = 0.891, SRMR = 0.070) fitted the data well. According to the principle of model parsimony suggested by Little (1997), we accepted the theoretical model as the most preferred model. The standardized coefficients for all paths estimated in the theoretical model are shown in Figure 2. Results showed that relational crafting was positively associated with work load (β = 0.146, *p* < 0.001), and that work load was positively associated with emotional exhaustion (β = 0.585, *p* < 0.001) after the influence of fixed control variables, indicating that Hypothesis 1b received support. However, the path coefficient between work load and work dynamics was not significant, demonstrating that Hypothesis 1a did not receive support. We held that the reason why the path coefficient between work load and work dynamics was negative but not significant might be that SSG and the control variables had a strong impact on work dynamics, enabling the effect of work load to be covered. Moreover, relational crafting was positively related to SSG (β = 0.188, *p* < 0.001), SSG was positively related to work dynamics (β = 0.331, *p* < 0.001), and that SSG was negatively related to emotional exhaustion (β = −0.127, *p* < 0.01) after the influence of fixed control variables (gender, age, education, and working years), indicating that Hypothesis 2a and Hypothesis 2b received support.

To test the mediation effect of work load and SSG, we used the procedures proposed by Preacher and Hayes (2004) and Preacher and Hayes (2008) and applied the bootstrapping method to further examine mediation through the “Process” plugin of SPSS 22.0. This method can produce higher statistical power. The bootstrapping sample size was set to 5,000, the confidence interval was set to 95%, and the results are shown in Table 3.

**TABLE 3:** Results of bootstrapping mediation effect examination.

Paths	Effect	S.E.	LLCI	ULCI
Relational crafting → work load → work dynamics	−0.009	0.008	−0.026	0.003
Relational crafting → work load → emotional exhaustion	0.087	0.056	0.021	0.195
Relational crafting → SSG → work dynamics	0.062	0.024	0.018	0.110
Relational crafting → SSG → emotional exhaustion	−0.023	0.015	−0.056	−0.008

The bootstrapping mediation analysis showed that, at the 95% confidence interval level, (1) the indirect effect of work load between relational crafting and work dynamics was −0.009, and the confidence interval (LLCI = −0.026, ULCI = 0.003) included 0, indicating that Hypothesis 1a did not get supported. (2) The indirect effect of work load between relational crafting and emotional exhaustion was 0.087, and the confidence interval (LLCI = 0.021, ULCI = 0.195) did not include 0, indicating that Hypothesis 1b was fully supported. (3) The indirect effect of SSG between relational crafting and work dynamics was 0.062, and the confidence interval (LLCI = 0.018, ULCI = 0.110) did not include 0, indicating that Hypothesis 2a was fully supported. (4) The indirect effect of SSG between relational crafting and emotional exhaustion was −0.023, and the confidence interval (LLCI = −0.056, ULCI = −0.008) did not include 0, indicating that Hypothesis 2b was fully supported.

## Discussion

The study was built on the JD-R model and the COR theory to examine how subordinates’ relational crafting impacted their job well-being. Our findings demonstrated that relational crafting had an opposite directional influence on job well-being through two different mediating variables (work load and SSG). More specifically, subordinates’ relational crafting harmed job well-being by increasing their work load but promoted their job well-being by enhancing SSG. Our findings indicate the complex mediating process of subordinates’ relational crafting on job well-being, presenting a more nuanced explanation of the relationship between relational crafting and job well-being. At the same time, we validated the JD-R model and the COR theory and shed light on two of their specific mechanisms before revealing both dysfunctional and functional outcomes of relational crafting.

### Theoretical Implications

First, the focus of our work extends the current job crafting literature by specifically examining relational crafting. As mentioned above, prior studies primarily try to recognize relational crafting as a type of job crafting and examine its effect (Slemp and Vella-Brodrick, 2014; Slemp et al., 2015; Lee and Lee, 2018). It is essential to understand the effects of relational crafting. Not only does it have unique characteristics different from task crafting and cognitive crafting, but it also has much greater significance in Chinese organizations with a high emphasis on *guanxi* (Li et al., 2018). Our study aimed to conduct an examination of subordinates’ relational crafting consequences and analyzed the potential for both negative and positive outcomes, which extended and updated the relevant studies.

Second, our study provides a new perspective to explore the beneficial mediator variables between subordinates’ relational crafting and attitude-related outcomes. Our study is the first to explicitly examine the mediating effect of SSG on relational crafting and job well-being. Prior job crafting literature attempts to test the positive consequences of intrinsic need, job autonomy, job engagement, job enjoyment, and team efficacy (Lee and Lee, 2018; Lazazzara et al., 2020). Most previous social network literature on SSG mainly focuses on the outcomes of SSG, including behavioral, attitudinal, and perceptual ones, such as job promotion, organizational commitment, and trust (Miao et al., 2020). We found that subordinates who craft their *guanxi* in the workplace are more likely to be categorized as “in-group” members by supervisors, thereby enhancing SSG and improving their job well-being. Thus, we found an antecedent variable of SSG and integrated two domains, namely, job crafting and social network, to contribute to job well-being.

Third, our study provides new evidence to understand the relationship between relational crafting and job well-being. In particular, we propose and prove that subordinates’ relational crafting has a double-edged sword effect on job well-being. Our results are consistent with those of Wrzesniewski and Dutton (2001), Gordon et al. (2015), Bruning and Campion (2018), and Lazazzara et al. (2020), who suggest that job crafting is not always positive. We extended this research concentrate on subordinates’ relational crafting and classified job well-being into work dynamics and emotional exhaustion, making a direct empirical examination of the important theoretical stipulation. As such, our work is significant for understanding the coexistence of the positive and negative sides of subordinates’ proactive behavior, as their relational crafting involves proactive self-initiating changes (Wrzesniewski and Dutton, 2001; Parker et al., 2010). The positive side of proactive behavior has been frequently discussed in prior literature, as shown in a meta-analysis by Thomas et al. (2011); nevertheless, most of the current studies did not capture the potential negative effect of proactive behavior. Our research showed that subordinates’ relational crafting can also negatively influence their job well-being through increased work load, which fills the gap and echoes the suggestion of Harju et al. (2021) to test the double-edged sword effect of job crafting.

Finally, our study takes a more unifying view to understand the complex mediating mechanism by integrating the JD-R model and the COR theory, in which resources are consumed or protected in the process of subordinates’ relational crafting. Although the beneficial mediator variables *via* the process of relational crafting positively influencing job well-being based on the self-determination theory and the JD-R model have been frequently investigated in prior literature (Tims et al., 2013; Slemp and Vella-Brodrick, 2014; Harju et al., 2021), the cost mediator variables *via* its negative influence on job well-being or the potential mediator variables that can explain the double-edged sword effect on job well-being are overlooked. Thus, we extended the existent research by exploring the resource loss path and the resource acquisition path of subordinates’ relational crafting in one model based on a new theoretical perspective, depicted as a double-edged sword effect on job well-being.

### Practical Implications

In addition to the theoretical implications, this study also provides guidance for practitioners. First, by providing evidence that relational crafting could improve job well-being, we hope to draw the attention of organizations and managers to the importance of carefully motivating and controlling subordinates’ relational crafting. On the one hand, organizations and managers should give subordinates opportunities to experiment with various behaviors of relational crafting. On the other hand, we raise the question of whether it is possible to develop a temperate level of subordinates’ relational crafting in which subordinates do not experience its major negative effect, the increased work load. Practically, we suggest that subordinates can adjust the development process of relational crafting according to the actual situation, as the process is a proactive behavior. For example, they could choose several measures, such as seeking external support and taking recovery activities, when perceiving demands that are beyond their capabilities and resources. Previous studies found that supervisory support reduces the negative effects of high job strain (Sargent and Terry, 2000), and recovery activities help employees’ resources return to pre-stress states (Kim S. et al., 2018). Meanwhile, we suggest that organizations and managers/supervisors may need to engage in several crafting training programs to better understand relational crafting and its potential negative side. Lee and Lee (2018) argued that with a better understanding of crafting, managers and supervisors can ensure positive outcomes. More importantly, managers/supervisors could take measures to reduce the negative effect of subordinates’ relational crafting on job well-being. For example, a relational crafting intervention may be considered as an effective measure to weaken the potential work load that might be caused by relational crafting (Van Wingerden et al., 2017). Furthermore, supervisors should provide subordinates (crafters) with adequate support and care to compensate for the time, energy, and cognitive resources consumed during relational crafting, as SSG is an important resource for amplifying work dynamics and buffering emotional exhaustion. Finally, as subordinates’ personal and job resources can be influenced by the working environment, and abundant resources can reduce the possibility of overload, organizations should create a supportive context, enriching their resources.

### Limitations and Future Research

Our study has several limitations. First, although the two-stage design reduces common method bias, the results also showed that this bias did not significantly affect our research, as the problems of the self-report questionnaire measures that we used in the empirical examination still exist (Hair et al., 2006). Accordingly, we suggest that future studies conduct a three-wave longitudinal study to analyze the mediation model more accurately. In addition, future studies could collect data from multiple and random sources because of the large Chinese population.

Second, we only examined the resource loss and resource acquisition perspectives *via* which relational crafting creates a double-edged sword effect based on the JD-R model and the COR theory. Future studies could test other possible mediating mechanisms, such as the self-presentational and the self-defense mechanisms that might explain the potential double-edged sword effect of relational crafting. Relational crafting can improve *guanxi* with supervisors and help employees gain their trust and information, and thus obtaining supervisors’ recognition. From the perspective of the self-presentational mechanism, in order to constantly meet supervisor’s expectations and demonstrate value, subordinates will be motivated to engage in more activities that are significant to the supervisor and the organization (Lau et al., 2014), resulting in increased work dynamics. However, the trust that subordinates gain from their supervisor through relational crafting often implies an increase in their own work-related obligations and an expansion of their role (Baer et al., 2015; Ren and Chadee, 2017). Based on the self-defense view, subordinates also perceive this trust as a potential threat that may increase their anxiety and emotional exhaustion.

Third, the current study confirmed that subordinates’ relational crafting had an opposite directional influence on job well-being through two paths. For a single path, we encourage researchers to conduct further discussions and examinations. For example, if relational crafting is poorly performed (e.g., by blindly pursuing one’s self-interest), it may also have negative effects, such as triggering colleagues’ jealousy, increasing excessive and unnecessary energy consumption, and increasing workload. In this way, the resource acquisition process may lead to decreased job well-being. Thus, we propose to define this impact as the too-much-of-a-good-thing effect of relational crafting and call for more research to test the issue.

Finally, Zhai et al. (2013) suggested that co-worker *guanxi* also needs to be labeled as a type of workplace *guanxi*, which inspired us to investigate the effect of relational crafting on both SSG and co-worker *guanxi*. In addition, Kim H. et al. (2018) found that task crafting can be costly (e.g., lower job satisfaction) to the crafter, providing thereby, some clues for further research. Future research may explore the double-edged-sword effect of other specific crafting forms, such as task crafting, and the mediating mechanisms.

## Data Availability Statement

The original contributions presented in the study are included in the article/supplementary material, further inquiries can be directed to the corresponding author.

## Author Contributions

SL predominantly contributed to conducting the literature review, designing the research, collecting some of the data, analyzing the data, and drafting the manuscript. BM and QW repeatedly revised and refined the content of the manuscript. QW contributed to helping to collect some of the data and drafting the manuscript. All authors substantially contributed to the research concept and design.

## Conflict of Interest

The authors declare that the research was conducted in the absence of any commercial or financial relationships that could be construed as a potential conflict of interest.

## Publisher’s Note

All claims expressed in this article are solely those of the authors and do not necessarily represent those of their affiliated organizations, or those of the publisher, the editors and the reviewers. Any product that may be evaluated in this article, or claim that may be made by its manufacturer, is not guaranteed or endorsed by the publisher.
